# Angiotensin II type 1 and type 2 receptor expression in circulating monocytes of diabetic and hypercholesterolemic patients over 3-month rosuvastatin treatment

**DOI:** 10.1186/1475-2840-11-153

**Published:** 2012-12-22

**Authors:** Franca Marino, Andrea Maria Maresca, Marco Cosentino, Luana Castiglioni, Emanuela Rasini, Christian Mongiardi, Ramona C Maio, Massimiliano Legnaro, Laura Schembri, Francesco Dentali, Anna Maria Grandi, Luigina Guasti

**Affiliations:** 1 Department of Clinical and Experimental Medicine, University of Insubria, Varese, Italy

**Keywords:** Type 2 Diabetes, Angiotensin II type 1 receptor, Angiotensin II type 2 receptor, Monocytes, Statin

## Abstract

**Background:**

In diabetes, a variety of pro-inflammatory cellular changes has been found in various cell types, including monocytes which are known to be involved in all the phases of atherogenesis. Angiotensin II (Ang II) type 1 receptor (AT_1_R) mediates the pro-atherogenic effects of Ang II whereas the type 2 receptor (AT_2_R) seems associated with atheroprotection. We sought to investigate the potential changes of AT_1_R-AT_2_R expression in human monocytes of type 2 diabetic- hypercholesterolemic patients and in hypercholesterolemic subjects, upon clinical treatment with rosuvastatin.

**Methods:**

The AT_1_R membrane protein and mRNA AT_1_R and AT_2_R expression in monocytes were investigated in 10 type 2 diabetic-hypercholesterolemic patients and in 10 hypercholesterolemic subjects, before and after 3-month rosuvastatin treatment. Moreover, the serum cytokine levels of interferon-γ (IFN-γ) and interleukin-4 (IL-4) were detected.

**Results:**

As expected, rosuvastatin was associated with a change in the lipid profile in the two groups. Both the membrane protein (*P* = 0.008) and the AT_1_R mRNA expression (*P* = 0.038) were significantly reduced during treatment in the absence of AT_2_R expression change in diabetic-hypercholesterolemic patients whereas no significant difference was observed in hypercholesterolemic subjects. The serum IL-4 levels were increased during treatment whereas no change was observed in IFN-γ in diabetic-hypercholesterolemic patients. No cytokine change was observed in hypercholesterolemic subjects.

**Conclusions:**

Our study on monocytes of diabetic-hypercholesterolemic patients, showing a reduced AT_1_R but not AT_2_R expression during rosuvastatin treatment, suggests that statin therapy may modulate favorably the AT_1_-AT_2_ receptor balance in subjects with coexistent type 2 diabetes.

## Background

Results from clinical trials show that systemic markers of inflammation correlate considerably with future cardiovascular events, both at baseline and during pharmacological treatments [1]. Atherosclerosis as an immuno-inflammatory disease has been associated with a prevalent T-helper (Th) 1 response, being the Interferon-γ (IFN-γ) a key cytokine for pro-inflammatory response amplification; although controversial, data support an antiatherogenic effect of Th2 responses, and Interleukin (IL)-4, the prototypic Th2-related cytokine, is generally considered as an anti-inflammatory cytokine [2]. The renin-angiotensin-system has been strongly implicated in atherogenesis-related pro-inflammatory events. Angiotensin II (Ang II) exerts its effects through the binding of two major receptors: the Ang II type-1 receptor (AT_1_R) which mediates the majority of the pro-atherogenic well-known Ang II actions and type-2 receptor (AT_2_R) that is considered counteracting the AT_1_R-mediated effects [3]. Recently a direct role of AT_1_R activation in leukocyte and macrophages has been identified in the persistence and/or amplification of microinflammation in vessel walls [4,5]. Although little is known at present about the involvement of AT_2_Rs in atherosclerosis, these receptors appear to participate importantly in vascular biology with improvement in resistance artery remodeling and appear to be cardioprotective [6-8].

The risk of atherosclerotic cardiovascular disease is increased 2- to 3-fold in type 2 diabetes mellitus and a recent meta-analysis in diabetic patients further strengthens the need of a clinical use of statins in this patient population, irrespectively from the baseline lipid characteristics of the patients [9]. Statins may interfere at various levels with the inflammatory processes leading to atherosclerosis and recent evidence, pointing to an interaction between statins and immune function, showed that isoprenoids could regulate T cell proliferation and Th1 differentiation [10,11]. Moreover, the effects of statins seem to influence pathways leading to Ang II-mediated atherosclerosis and we previously demonstrated, in circulating neutrophils of high-cardiovascular risk patients, interferences of treatment on both AT_1_R expression and the cell membrane translocation Rac 1, a guanosine triphosphate–binding protein playing a key role in Ang II–operated signaling pathways [12].

Among the circulating immune cells, monocytes are the most studied cell subset in relation to atherosclerosis and a key role of these cells in all the phases of atherogenesis has been demonstrated. Monocytes are the primary immune cells that appear early on the endothelial layer and adhere to the arterial endothelium and, after migrating into the intima and accumulating in fatty streaks, contribute to their differentiation into macrophages which produce inflammatory cytokines, stimulate smooth muscle cell proliferation and migration and produce proteolytic enzymes that render the growing plaque more susceptible to rupture [13]. In the attempt to dissect the pathophysiological aspects and causalities of atherosclerosis and the potential effects of pharmacological treatments, it is worth focusing on cellular and subcellular pro-atherogenetic changes associated with atherosclerosis and their modulation by drugs. However, most of the reported studies on the so-called pleiotropic effects of statins have been performed in vitro or in animal models of atherosclerosis [14,15] and only very few studies are available on cellular effects of clinically administered statin treatment in humans. Recently a word of caution has been raised on the basis of the notion that the various mouse models in use for studying atherosclerosis differ noticeably in their response to pharmacological modifiers of atherogenesis and differences in anatomy, lipid metabolism, and gene expression complicate translation of experimental results obtained in mice to humans [16].

Moreover, a challenge of the human cellular studies in atherosclerosis relies on the evidence that atherosclerosis is a highly heterogeneous condition. To minimize this limitation of human studies, we focused on diabetic patients who did not show clinical manifestation of atherosclerosis, such as healthy subjects with pre-clinical and early pro-atherogenous changes. We have previously demonstrated that polymorphonuclear cells and monocytes obtained from high-risk subjects express more AT_1_R than monocytes from healthy donors [17-19]. In the present study we sought to investigate whether rosuvastatin treatment in diabetic-hypercholesterolemic patients could be associated with a greater modulation of AT_1_R and AT_2_R expression in monocytes than in only hypercholesterolemic subjects treated with rosuvastatin. Moreover, we investigated whether the statin treatment could interfere with Th1/Th2 profile as expressed by the measure of serum IFN-γ and IL-4 levels.

## Patients and methods

### Design of the study

In 10 subjects with type 2 diabetes and hypercholesterolemia (DM), and in 10 hypercholesterolemic (HC) subjects, monocytes were isolated from venous blood to investigate the AT_1_R and AT_2_R expression; moreover serum was also collected to measure cytokine production (IFN-γ, IL-4) (see below). Patients were studied: 1] before any pharmacological treatment and 2] after 3- month treatment with rosuvastatin (10 mg/day; 10 PM). The subjects were enrolled consecutively at our Lipid Clinic (Research Center on Dyslipidemia, Clinical Medicine, University of Insubria, Varese, Italy) coming from the Diabetic Unit or General Practitioner for evaluation of dyslipidemia. As for inclusion criteria to be enrolled in this study, no disease was found after a clinical examination and routine laboratory tests (apart from diabetes), and the patients did not assume any pharmacological treatment except for anti-diabetic treatment. Anti-diabetic medications were allowed if the patients were satisfactorily treated and no changes in treatment were scheduled for the duration of the study. Eight patients were on diet treatment, 1 assumed metformin and 1 patient was on treatment with the association glybenclamide/metformin. Ongoing clinical infection and/or the presence of infections in the previous three months as well as a smoking *habitus* and competitive sporting activities were exclusion criteria. All the patients had previously been instructed about the life-style modification required for the treatment of their disease, including dietary treatment (qualitative counseling) and mild physical activity. The patients were then asked to maintain the same level of physical activity and a similar diet throughout the study. All the patients were evaluated with a clinical visit including familial and personal history. Among the diabetic-hypercholesterolemic patients studied, 8 were males, mean age was 68 ± 8 years whereas among hypercholesterolemic subjects mean age was 65 ± 10 years. The body-mass-index (28.4 ± 2.7 and 27.4 ± 2.8 Kg/m^2^) and waist circumference (102.6 ± 10.4 and 99 ± 10cm) were similar respectively in diabetic and hypercholesterolemic patients and the values did not change throughout the study. Blood samples were obtained to perform routine laboratory exams and to isolate circulating monocytes for subsequent studies and to measure serum cytokines by using heparinized tubes between 8.00 and 9.00 A.M., after a night of fasting. Glomerular filtration rate was measured according to MDRD formula [20]. All the subjects were previously asked not to take coffees, teas, chocolates, or cola-containing substances for the 24 hours preceding the evaluations. Our study complies with the Declaration of Helsinki; the local Ethics Committee has approved the research protocol and informed consent has been obtained from the subjects.

### Cell isolation

Whole blood was allowed to sediment on dextran at 37°C for 30 min. Supernatant was recovered and peripheral blood mononuclear cells (PBMCs) were separated by Ficoll-Paque Plus density-gradient centrifugation. A typical PBMC preparation contained about 80% lymphocytes and 16% monocytes, and cell viability was always >99% as assessed by flow cytometric analysis.

Monocytes (for real-time evaluation) were further separated from PBMCs by immunomagnetic cell sorting. To this end, antibodies targeted to CD14 (monocytes) were obtained from Dynal A.S. (Oslo, Norway) and added to separate aliquots of the cell suspension using a target-to-bead ratio of 1:4 as previously described [17].

### Flow cytometric analysis of AT_1_R expression

We have evaluated the expression of AT_1_Rs on the cell membrane of monocytes. To this end, 1 ml of whole blood was used and the analysis was performed by using conventional immunofluorescence techniques together with a multiparametric flow cytometric analysis as previously described [17]. A minimum of 50.000 cells were analyzed from each sample, and AT_1_R density on positive cells [mean fluorescence intensity (MFI)] was obtained.

### RNA isolation and real-time polymerase chair reaction (PCR) analysis of AT_1_R and AT_2_R mRNA

Total mRNA was extracted from 1x10^6^ monocytic cells by Perfect RNA Eukaryotic Mini kit (Eppendorf, Hamburg, Germany) and the amount of extracted RNA was estimated by spectrophotometry at 260 nm. Total RNA was reverse transcribed using the high-capacity cDNA Archive Kit (Applied Biosystems, Foster City, USA) according to the manufacturer’s instructions.

Real-time PCR was performed by means of an ABI prism 7000 apparatus (Applied Biosystem, Foster City, CA) using the assay-on-demand kits (Table 1) as previously described [17,21].

**Table 1:** Real-time-Protein Chain Reaction primers

**Gene name**	**Interrogated sequence**		**Translated protein**	**Exon boundary**	**Assay location**	**IMAGE clone ID**
**AT**_**1**_**R**	RefSeq	NM_031850.1	NP_114038.1	1-2	248	-
		NM_000685.3	NP_000676.1	1-2	248	-
	GeneBank mRNA	S77410.1	-	1-2	201	-
		BC068494.1	-	1-2	224	30337806
**AT**_**2**_**R**	RefSeq	NM_000686.3	NP_000677.2	2-3	129	-
	GeneBank mRNA	U16957.1	-	2-3	107	-

AT_1_R = Angiotensin II type 1 receptor; AT_2_R = Angiotensin II type 2 receptor.

Threshold cycle values (Ct1) for the genes of interest were calculated, normalized to 18s RNA (Ct2) (housekeeping) content and finally expressed as 2^-ΔCt^, where ΔCt = Ct2 - Ct1.

### Measurement of serum cytokines

Serum samples collected were analysed for cytokine content. To this end, IFN-γ, IL-4 levels in serum were quantified using a sandwich-type enzyme-linked immunosorbent assay (ELISA kit; Amersham Biosciences, UK). The detection limit of the assay was 1 pg/ml. The control values for serum IL-4 levels were: not detectable; and the serum control IFN-γ levels were: 0–1.5 pg/ml, as reported for the kit used.

### Statistical analysis

Data are presented as mean ± standard deviation (SD). A paired *t* test was used to compare variables before and during pharmacological statin treatment. Calculations were performed using a commercial software (GraphPad Prism version 5.00 for Windows, GraphPad Software, San Diego, CA, USA, http://www.graphpad.com) and a two-sided *P* < 0.05 was retained for statistical significance.

## Results

Lipid pattern and other laboratory characteristics were not different at baseline in the two groups (Additional file 1). As expected and shown in Table 2, rosuvastatin treatment significantly affected the lipid profile of the patients after the 3-month treatment. No change was observed in liver and renal function. High-sensitive serum C-reactive protein was also reduced after statin treatment. 

**Table 2:** Laboratory characteristics of patients at baseline and after three-month rosuvastatin therapy

	** Diabetic patients (n = 10)**			** Hypercholesterolemic patients (n = 10)**		
	**baseline**	**treatment**	**#P**	**baseline**	**treatment**	**#P**
TC (mg/dl)	223.7 ± 34.0	168.5 ± 45.9	0.003	288.2 ± 41.0	165.5 ± 24.4	<0.001
TG (mg/dl)	165.3 ± 73.0	118.5 ± 31.5	0.012	180.9 ± 61.4	133.5 ± 58.9	0.004
HDL-c (mg/dl)	50.0 ± 2.8	50.2 ± 6.5	0.94	55.1 ± 9.9	50.7 ± 12.1	0.164
LDL-c (mg/dl)	135.7 ± 20.6	85.0 ± 25.6	<0.001	197.0 ± 34.1	88.1 ± 25.1	<0.001
ApoA (mg/dl)	128.0 ± 53.0	163.0 ± 14.4	0.69	144.5 ± 28.5	160.1 ± 55.4	0.34
ApoB (mg/dl)	117.2 ± 29.1	54.0 ± 14.9	<0.001	147.1 ± 38.8	75.1 ± 24.6	<0.001
Glucose (mg/dl)	137.7 ± 19.4	135.5 ± 31.8	0.79	98.6 ± 5.7	97.0 ± 12.9	0.77
Insulinemia (ng/ml)	14.2 ± 3.4	16.3 ± 3.5	0.63	18.8 ± 14.7	19.7 ± 17.0	0.63
AST	24.2 ± 4.3	24.0 ± 4.1	0.94	18.3 ± 5.6	17.1 ± 3.5	0.658
ALT	37.7 ± 19.2	39.2 ± 27.1	0.78	27.6 ± 12.9	21.3 ± 4.4	0.122
GGT	43.3 ± 27.2	46.5 ± 39.6	0.65	21.7 ± 5.9	20.1 ± 5.6	0.131
CK	226.8 ±113.7	212.7 ± 103.5	0.73	135.7 ± 64.9	134.4 ± 56.6	0.994
s-creatinine	0.98 ± 0.11	0.97 ± 0.10	0.89	0.98 ± 0.17	0.99 ± 0.18	0.597
GFR	76.33 ± 12.18	77.5 ± 3.56	0.79	73.22 ± 33.31	74.00 ± 12.09	0.667
hsCRP	2.94 ± 2.02	1.76 ± 0.51	0.03	1.70 ± 1.69	1.15 ± 0.79	0.350

TC = total cholesterol; TG = triglycerides; HDL-c = high density lipoprotein-cholesterol; LDL-c = low density lipoprotein-cholesterol; ApoA = Apolipoprotein A; ApoB = Apolipoprotein B; AST = aspartate aminotransferase; ALT = alanine aminotransferase; GGT = γ-glutamyl transpeptidase; CK = creatine kinase; GFR = glomerular filtration rate; hsCRP = high sensitivity C reactive protein. # Paired *t* test.

### Flow cytometric analysis of AT_1_R expression

**Figure 1:**
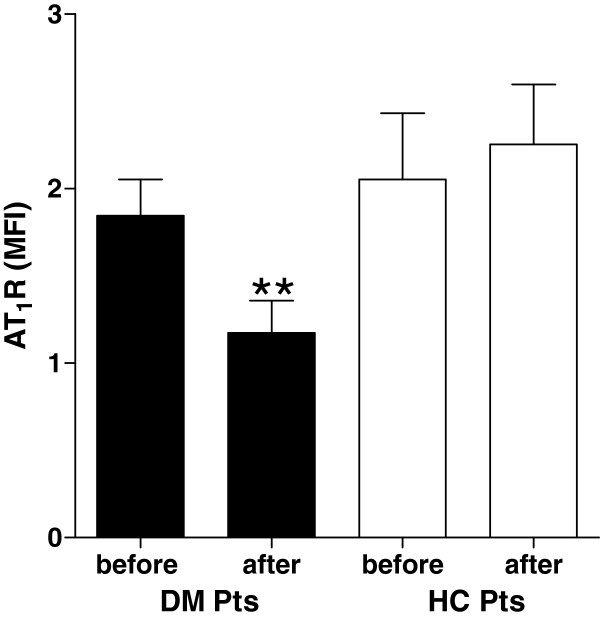
**Expression of AT**_**1**_**R in diabetic and hypercholesterolemic subjects before and after 3 months of rosuvastatin treatment.** Legend: DM pts, diabetic patient; HC pts, hypercholesterolemic patients.** *P = 0,008* vs values obtained before statin treatment.

Figure 1 shows data of a representative flow cytometric analysis of AT_1_R expression on monocytes of a diabetic subject obtained before and after statin treatment (panel A). The data shown are obtained from one representative subject. A 3-month rosuvastatin treatment significantly reduced AT_1_R membrane expression in monocytes of diabetic patients. Values (measured as MFI) observed before the institution of therapy were 1.84 ± 0.55 and reached the values of 1.17 ± 0.45 after the clinical statin treatment (*P* = 0.008). Moreover in hypercholesterolemic subjects we didn't see any difference (panel B).

### AT_1_R and AT_2_R mRNA expression in monocytes from patients before and after rosuvastatin treatment

**Figure 2:**
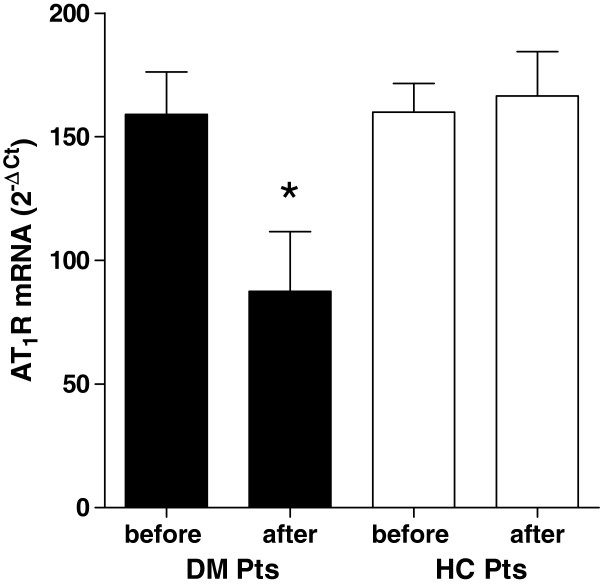
**AT**_**1**_**R mRNA levels in monocytes of diabetic and hypercholesterolemic subjects before and after 3 months of rosuvastatin treatment.** Legend: DM pts, diabetic patient; HC pts, hypercholesterolemic patients. * *P* < 0.05 vs values obtained before statin treatment.

**Figure 3:**
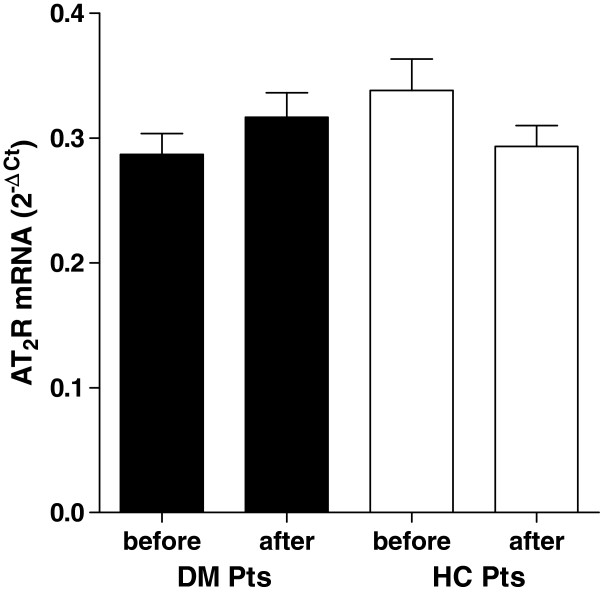
**AT**_**2**_**R mRNA levels in monocytes of diabetic and hypercholesterolemic subjects before and after 3 months of rosuvastatin treatment.** Legend: DM pts, diabetic patient; HC pts, hypercholesterolemic patients.

As observed for the protein expression, the mRNA expression of AT_1_R was significantly reduced during treatment in monocytes of diabetic patients (Figure 2, panel A) whereas no difference was observed for the AT_2_R expression measured before and during statin therapy (Figure 3, panel A).

No differences was observed for AT_1_R and AT_2_R expression measured before and during therapy in hypercholesterolemic subjects (Figures 2 and 3, panel B).

### Serum cytokine levels from patients before and after rosuvastatin treatment

Rosuvastatin treatment did not affect serum levels of IFN-γ (7.97 ± 2.41 pg/ml *vs* 6.45 ± 3.35 pg/ml; *P* = 0.912). A significant increase of IL-4 was observed after three months of statin treatment (22.96 ± 16.53 pg/ml *vs* 35.10 ± 16.93 pg/ml; *P* = 0.020). No cytokine change was observed in hypercholesterolemic subjects.

## Discussion

The main finding of the present study is the observation that medium-term rosuvastatin clinical treatment is associated with reduced AT_1_R expression in monocytes of type 2 diabetic patients in the absence of AT_2_R expression changes.

Various factors act in diabetes to induce atherosclerotic processes and vascular changes. Both hyperinsulinemia and the lipid pattern called atherogenic dyslipidemia are well known pro-atherogenic factors [22]. Recently, the central role of inflammation in diabetes-related atherosclerosis has been underlined by the finding that pro-inflammatory preconditioning is required for the development of high-glucose-induced inflammation in human aortic smooth muscle cells [23]. Moreover, altered cellular functions of various cell types have been related to diabetes and these abnormalities may contribute to systemic inflammation. In particular, in experimental models of diabetes monocytes/macrophages show a decreased phagocytosis and an increased expression of inflammatory mediators, and cells from diabetic subjects exhibit enhanced adherence to the endothelium [24]. As regards the Ang II receptor expression, the functional relevance of up-regulated AT_1_R expression in diabetes has been underlined by a recent study showing that upregulation of the ligand–Receptor for Advanced Glycation End products pathway via AT_1_R is an essential mechanism in diabetic atherosclerosis [25]. In diabetic myocardium a significant increase in protein expression and median mRNA expression of the AT_1_R was reported [26]. In carotid human specimen the mRNA expression of both angiotensinogen and angiotensin-converting enzyme were higher in type 2 diabetic patients than in non-diabetic subjects whereas AT_1_R mRNA did not differ [27]. Previously we reported that subjects with increased cardiovascular risk according to the Adults Treatment Panel III guidelines had an increased leukocyte AT_1_R expression at mRNA level and that this expression was reduced during simvastatin treatment but no study has previously focused on cellular changes upon statin treatment in a specific diabetic population [12,17]. In this study we did not enrolled a control group since the comparison of the receptor expression of high risk subjects has been already investigated [17]. Moreover, we show here for the first time that not only mRNA, but also protein membrane expression of AT_1_R was significantly reduced during rosuvastatin treatment.

AT_2_Rs are present in the vasculature and may induce vasorelaxation in vivo [6-8]. Very few studies are at present published on the AT_2_R expression and functioning in humans, and it is now generally accepted that the AT_1_R blockade is associated with AT_2_R overexpression and that AT_2_R stimulation by Ang II induces counterregulatory vasodilatation that opposes AT_1_R–mediated vasoconstriction [28]. Indeed, in a study on resistance arteries dissected from gluteal subcutaneous tissues of hypertensive diabetic patients treated with valsartan, Ang II evoked a significant vasodilatory response which was blocked by an AT_2_R inhibitor [6]. However, studies performed on human internal mammary arteries, AT_2_R receptor stimulation did not mediate vasodilation and AT_2_R-mediated vasodilation in the human heart was shown to be limited to coronary microarteries [29,30].

To our knowledge, no study is available on both AT_1_R and AT_2_R modulation by rosuvastatin. In this study, we show for the first time that, after treatment with rosuvastatin, no changes are observed in AT_2_R mRNA expression in monocytes of type 2 diabetic patients. This finding strengthens the results of reduced AT_1_R observed during treatment, suggesting a possible favorable modulation of the AT_1_-AT_2_ receptor expression during statin treatment. Moreover, according with the already reported finding of a relatively lower AT_2_R tissue expression in the adult when compared with AT_1_R expression [28], we show in human monocytes of diabetic subjects that the AT_2_R is expressed at a lower level than AT_1_R. However we have to acknowledge that the results reported here, obtained in a population mainly constituted by male subjects, apply only to type 2 diabetic patients. Even if we have not recruited a control group, the reduction of AT1 receptor expression after rosuvastatin treatment could be explained by a specific effect of diabetes, given that the two groups didn’t differ in their lipid profile. Moreover, the relatively small number of the patients enrolled in this study or the short-term could have influenced the results in hypercholesterolemic patients.

It has been recently reported that rosuvastatin was able to affect the Th1/Th2 response in humans with acute coronary syndrome [31]. In our study, in subjects prone to atherosclerosis but in a stable phase of the process as the type 2 diabetic patients studied, a significant increase of the Th2-profile-related IL-4 was observed whereas we did not evidence a reduction in circulating IFN-γ during treatment. Previously, a reduction in pro-inflammatory cytokine production by circulating monocytes has been associated with statin treatment in humans [32,33]. As regards statin effects on cytokines directly involved in immunomodulation, although simvastatin-treated dendritic cells showed a Th2 transcription profile which was accompanied by increased Th2 (IL-4, IL-5, and IL-13) and decreased Th1 (IFN-γ) cytokine secretion from the T cells [10], no longitudinal study in humans has previously shown increased IL-4 levels during clinical statin treatment. Other authors showed no immunomodulatory effect by atorvastatin on the Th1/Th2 balance in human T cells in vitro [34].

## Conclusions

In conclusion, this longitudinal study in humans show that rosuvastatin treatment is associated with reduced monocyte AT_1_R but not AT_2_R expression, suggesting that the clinically administered statin therapy may modulate favorably the AT_1_-AT_2_ receptor balance in diabetic patients.

## Abbreviations

Ang II: Angiotensin II; AT_1_R: Angiotensin II type 1 receptor; AT_2_R: Angiotensin II type 2 receptor; IFN-γ: Interferon-γ; IL: Interleukin; Th: T-helper; PBMCs: Peripheral blood mononuclear cells; MFI: Mean fluorescence intensity; SD: Standard deviation.

## Competing interests

The authors declare that they have no competing interests

## Authors' contributions

FM, AMM and LG: design, data collection, drawing the manuscript, data analysis and statistics. All authors: design, critical revision of article and approval of article.

## Supplementary Material

Additional file 1 Laboratory characteristics of patients at baseline. Click here for file
