# Supplementary material for: Angiotensin II type 1 and type 2 receptor expression in circulating monocytes of diabetic and hypercholesterolemic patients over 3-month rosuvastatin treatment
Source: Cardiovasc Diabetol. 2012 Dec 22;11:153. doi: 10.1186/1475-2840-11-153 (PMC3549933; doi:10.1186/1475-2840-11-153)
Supplement: Additional file 1 — Laboratory characteristics of patients at baseline. [file 1475-2840-11-153-S1.doc]

**Supplementary data.** Laboratory characteristics of patients at baseline

Diabetic patients Hypercholesterolemic patients P

(n=10) (n=10)

| TC (mg/dl) | 223.7 ± 34.0 | 288.2 ± 41.0 | 0.243 |
| --- | --- | --- | --- |
| TG (mg/dl) | 165.3 ± 73.0 | 180.9 ± 61.4 | 0,872 |
| HDL-c (mg/dl) | 50.0 ± 2.8 | 55.1 ± 9.9 | 0,626 |
| LDL-c (mg/dl) | 135.7 ± 20.6 | 197.0 ± 34.1 | 0,141 |
| ApoA (mg/dl) | 128.0 ± 53.0 | 144.5 ± 28.5 | 0,787 |
| ApoB (mg/dl) | 117.2 ± 29.1 | 147.1 ± 38.8 | 0,545 |
| Glucose (mg/dl) | 137.7 ± 19.4 | 98.6 ± 5.7 | 0,069 |
| Insulin (ng/ml) | 14.2 ± 3.4 | 18.8 ± 14.7 | 0,764 |
| AST | 24.2 ± 4.3 | 18.3 ± 5.6 | 0,414 |
| ALT | 37.7 ± 19.2 | 27.6 ± 12.9 | 0,668 |
| GGT | 43.3 ± 27.2 | 21.7 ± 5.9 | 0,448 |
| CK | 226.8 ±113.7 | 135.7 ± 64.9 | 0,495 |
| s-creatinine | 0.98 ± 0.11 | 0.98 ± 0.17 | 1,000 |
| GFR | 76.33 ± 12.18 | 73.22±33.31 | 0,931 |
| hsCRP | 2.94 ± 2.02 | 1.70 ± 1.69 | 0,643 |

TC = total cholesterol; TG = triglycerides; HDL-c = high density lipoprotein-cholesterol; LDL-c = low density lipoprotein-cholesterol; ApoA = Apolipoprotein A; ApoB = Apolipoprotein B; AST = aspartate aminotransferase; ALT = alanine aminotransferase; GGT = γ-glutamyl transpeptidase; CK = creatine kinase; GFR = glomerular filtration rate; hsCRP = high sensitivity C reactive protein. # Paired t test.
